# Strength and size of phosphorus-rich patches determine the foraging strategy of *Neyraudia reynaudiana*

**DOI:** 10.1186/s12870-020-02738-0

**Published:** 2020-12-07

**Authors:** Liping Cai, Yuzhen Wang, Mulualem Tigabu, Xiaolong Hou, Pengfei Wu, Chuifan Zhou, Xiangqing Ma

**Affiliations:** 1 College of Forestry, Fujian Agriculture and Forestry University, Fuzhou, 350002 China; 2 Key Laboratory of State Forestry Administration for Soil and Water Conservation in Red Soil Region of China, Fuzhou, 350002 China; 3 Cross-Strait Collaborative Innovation Center of Soil and Water Conservation, Fuzhou, 350002 China; 4 Han Dan NO.2 Foreign Language School, Handan, 056000 China; 5 Southern Swedish Forest Center, Swedish University of Agricultural Sciences, Box 49, Sundsvägen 3, 23053 Alnarp, Sweden

**Keywords:** Nutrient foraging, Nutrient patches, Root morphological plasticity, Root physiological plasticity, Phosphorus stress

## Abstract

**Background:**

Under natural conditions, soil nutrients are heterogeneously distributed, and plants have developed adaptation strategies to efficiently forage patchily distributed nutrient. Most previous studies examined either patch strength or patch size separately and focused mainly on root morphological plasticity (increased root proliferation in nutrient-rich patch), thus the effects of both patch strength and size on morphological and physiological plasticity are not well understood. In this study, we examined the foraging strategy of *Neyraudia reynaudiana* (Kunth) Keng ex Hithc, a pioneer grass colonizing degraded sites, with respect to patch strength and size in heterogeneously distributed phosphorus (P), and how foraging patchily distributed P affects total plant biomass production. Plants were grown in sand-culture pots divided into ½, ¼, 1/6 compartments and full size and supplied with 0 + 0/30, 0 + 7.5/30 and 7.5 + 0/30 mg P/kg dry soil as KH_2_PO_4_ or 0 + 15/15, 0 + 18.5/ 18.5, 7.5 + 15/15 mg kg − 1 in the homogenous treatment. The first amount was the P concentration in the central region, and that the second amount was the P concentration in the outer parts of the pot.

**Results:**

After 3 months of growth under experimental conditions, significantly (*p* < 0.05) high root elongation, root surface area, root volume and average root diameter was observed in large patches with high patch strength. Roots absorbed significantly more P in P-replete than P-deficient patches. Whole plant biomass production was significantly higher in larger patches with high patch strength than small patches and homogeneous P distribution.

**Conclusion:**

The result demonstrates that root morphological and physiological plasticity are important adaptive strategies for foraging patchily distributed P and the former is largely determined by patch strength and size. The results also establish that foraging patchily distributed P resulted in increased total plant biomass production compared to homogeneous P distribution.

**Supplementary Information:**

The online version contains supplementary material available at 10.1186/s12870-020-02738-0.

## Background

Under natural conditions, soil nutrients are heterogeneously distributed and patches with different sizes, nutrient availability and nutrient content are mosaic in space [1]. Spatio-temporal variations in litter inputs, decomposition and subsequent release of nutrients are among the causes of patchily distribution of nutrient in the soil [2]. Evidence shows that spatial variability in nutrient availability in soils occurs within ranges reachable by most plant roots [3, 4]. To forage localized soil nutrient patches, plants have evolved several adaptive mechanisms; including deployment of more roots in nutrient-rich patches – the so called root morphological plasticity [5, 6]. Most plants forage nutrients in the heterogeneous space through proliferation of lateral roots, increased root biomass, root length, root surface area and root volume in nutrient-rich patches; thereby regulating the contact range and area of root-soil [7, 8]. Another mechanism by which plants forage nutrient in heterogeneous environment is through increasing nutrient uptake rates in nutrient-rich patches – the so called physiological plasticity [6, 9]. In addition, heterogeneous nutrient supply may enhance growth and biomass production, although the effect varies among species [10, 11].

Nutrient foraging in spatially heterogeneous environment is not only related to plant species, but also affected by nutrient patch attributes, nutrient elements and overall nutrient supply. For instance, significant differences in root proliferation in response to nitrate supply was observed between *Lupinus angustifolius* and *Lupinus pilosus* [12]. It has also been demonstrated that plants do not need root hyperplasia to acquire N in heterogeneous patches while they acquire the difficult-to-move phosphorus (P) by root hyperplasia [13]. However, the experiment on *Agropyron desertorum* showed opposite results that the new roots are responsive to N patches but non-responsive to P patches [14]. Whereas the fine roots of *Picea sitchensis* seedlings were significantly proliferated in the NO_3_^−^, NH_4_^+^ or P nutrient patches [15]. These studies demonstrate that generalization of root responses to patchily distributed nutrients is still difficult to make and the responses are species-specific.

The efficacy of root plasticity is also mainly determined by the strength and size of the nutrient-replete patch [16, 17]. Patch strength is defined as the difference in nutrient concentration between neighboring patches, and patches with high strength have a large nutrient concentration compared with the adjacent patches. Most previous studies investigated root proliferation in response to patch strength; i.e. the concentration gradient between nutrient-rich and nutrient-poor patches [8]. However, few studies have examined root morphological responses to varying patch strength and patch size simultaneously [11, 17, 18]. Therefore, empirical evidence on how patch strength and patch size influence the root morphological and physiological plasticity, thereby enhancing nutrient foraging in heterogeneous nutrient environment, is still scarce.

In this study, we demonstrated the effects of patch strength and patch size on root morphological and physiological plasticity using *Neyraudia reynaudiana* (Kunth) Keng ex Hithc as a model plant. *N. reynaudiana* is a fascinating perennial grass species that can grow in highly degraded and barren land such as rock outcrops and in mine wastelands [19]. As a result, it is widely planted on degraded sites in East Asia for soil and water conservation purposes [20]. Previous studies focused on understanding the mechanisms by which this species cope up with low nutrient stress and thrives well on degraded sites, including high nitrogenase activities in the stems [21], symbiotic relationship between roots of *N. reynaudiana* and VA mycorrhizal fungi [22] and phosphate-solubilizing fungi [23], increased activities of POD, SOD and CAT enzymes and increased soluble protein content in leaves and acidic root exudates under low nutrient stress [24] and changes in root morphological traits in response to low P supply [8, 24]. These studies had, however, been made under homogeneous low nutrient environment or under different patch strength levels only in heterogeneous P environment [8]. Foraging strategy in response to patch mosaics with different sizes and strengths has not been studied in this species. An understanding of the foraging strategy is a key to unravel the adaptation mechanisms of *N. reynaudiana* to extremely harsh environment and efficient use of the meager resources.

The main objective was to examine the effects of patch size and patch strength on root morphological traits, root P contents and physiological plasticity and whole-plant biomass production of *N. reynaudiana*. The study addressed the following research questions: (1) is root proliferation in P-rich patches influenced by patch size and patch strength?; i.e. is root morphological plasticity a major foraging strategy to patchily distributed P?; (2) does root P content vary with patch size and strength?, and does physiological plasticity play an adaptive role in foraging patchily distributed P?; and (3) is total plant biomass production higher in heterogeneous than homogenous P distribution condition, and if so is it related to patch size and patch strength? We hypothesized that (1) *N. reynaudiana* deploys more roots in P-replete patches with higher patch strength and large patch size than in patches with low patch strength and small patch size; (2) root P content will be higher in roots grown in P-replete than P-deficient patches and varies with patch strength and patch size, thus physiological plasticity plays an important adaptive role (3) total plant biomass production is expected to be higher in heterogeneous than homogeneous P distribution and varies with patch strength and patch size in heterogeneous distribution due to increased uptake of nutrients.

## Results

### Selective deployment of roots in spatially heterogeneous P distribution over time

**Fig. 1:**
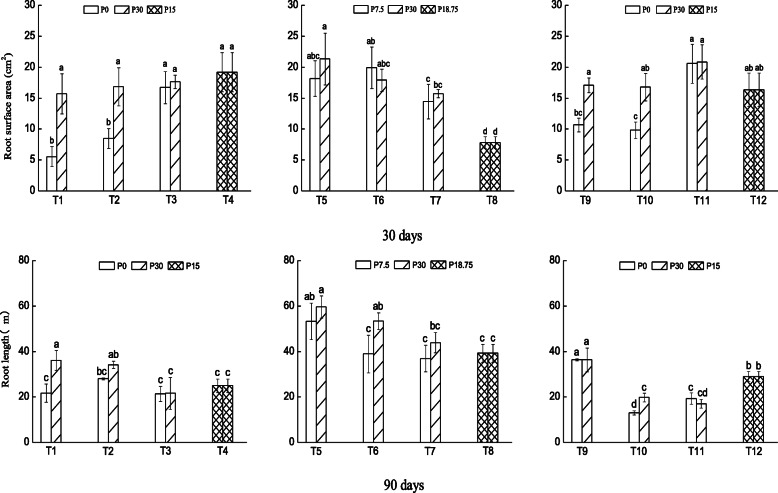
Total root length (m) of *N. reynaudiana* grown in high and low P patches under heterogeneous and homogenous P distribution for 30 (top panel), and 90 days (bottom panel). T1, T2 and T3 had high P concentration gradient between patches (0 and 30 mg.kg^− 1^), T5, T6 and T7 had moderate P concentration gradient (7.5 and 30 mg kg^− 1^), T9, T10 and T11 had initial P concentration of 7.5 mg kg^− 1^ in the central interior patch; i.e. P-enriched patch, and subsequently high P concentration gradient between patches (0 and 30 mg kg^− 1^); and T4, T8 and T12 had homogenous P distribution of 15, 18.5 and 7.5 + 15 mg kg^− 1^, respectively. Values are mean ± SE (*n* = 3), * indicates significant difference at *p* < 0.05 and lowercase letters indicate significant difference between treatments

There were statistically significant differences in the combined root morphological traits in response to patch strength, patch size, compartments (high P versus low P) and their interactions over time (Table 1). When the root morphological traits were considered separately, no significant effects of patch strength × patch size on total root length and patch size × compartment on root length and root volume were detected. Generally, all root morphological traits increased over time from 171 cm after 30 days of growth to 3285 cm after 90 days in total root length, from 16 cm^2^ after 30 days to 391 cm^2^ after 90 days in root surface area, from 0.3 mm after 30 days to 0.6 mm after 90 days in average root diameter and 0.12 cm^3^ after 30 days to 2.67 cm^3^ after 90 days in root volume. The total root length and average root diameter increased with decreasing patch strength while root surface area and root volume increased with increasing patch strength across all levels of patch size. Across all levels of patch strength, all root morphological traits increased with increasing patch size. Total root length after 30 days of growth was higher in large and medium patches with high P than low P when the patch strength was high with or without initial encounter with P-enriched patch (Fig. 1 top panel; T1–T2; T9–10). Moderate patch strength generally favored root elongation in large patches compared to medium and small patches (Fig. 1 top panel; T5–T8). Root elongation after 60 days of growth was still high in P-replete large patches while the opposite was observed in medium patches and no difference could be discerned in small patches when the patch strength was high (Supplementary material S1). After 90 days of growth, root elongation was still high in P-replete large patch when the patch strength was high, in P-replete medium patch when the patch strength was moderate and when plants grown initially in P-enriched patch followed by high patch strength (Fig. 1 bottom panel; T1, T6, T10).

**Table 1:** Results of multivariate ANOVA to examine the effects of patch strength and patch size on root morphological traits in low P and high P compartments

Source	Wilk’s Lambda	d.f	F	***p***
Patch strength (S)	0.01	10	84.9	0.0001
Patch size (Ps)	0.02	15	28.8	0.0001
Compartment (C)	0.51	5	8.6	0.0001
Time (T)	0.01	8	168.5	0.0001
S × Ps	0.02	30	10.9	0.0001
S × C	0.44	10	4.5	0.0001
Ps × C	0.37	15	3.5	0.001
S × T	0.024	16	47.72	0.001
Ps × T	0.136	16	18.55	0.001
C × T	0.646	8	6.41	0.001
S × Ps × C	0.34	30	1.9	0.007
S × Ps × T	0.086	32	11.51	0.007
S × C × T	0.425	16	6.50	0.001
Ps × C × T	0.577	16	3.97	0.001
S × Ps × C × T	0.271	32	20.84	0.001

**Fig. 2:**
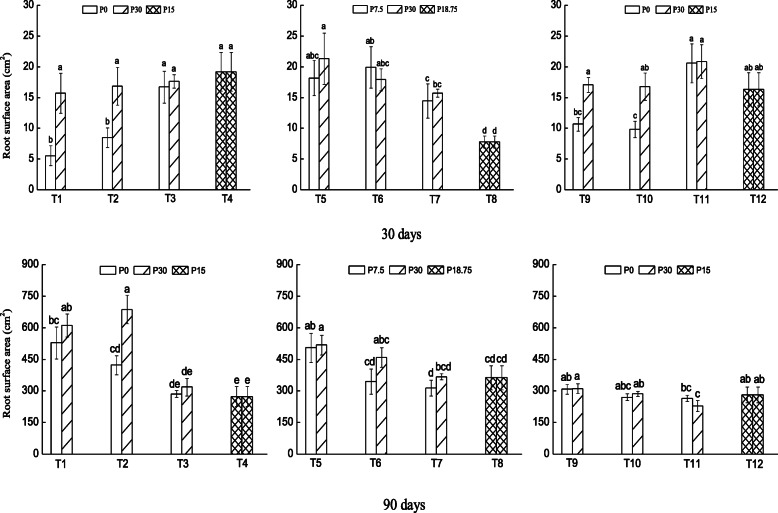
Root surface area (cm^2^) of *N. reynaudiana* grown in high and low P patches under heterogeneous and homogenous P distribution for 30 (top panel) and 90 days (bottom panel). Treatments, T1 – T12, are as described in Fig. 1 and values are mean ± SE (*n* = 3), * indicates significant difference at *p* < 0.05 and lowercase letters indicate significant difference between treatments

After 30 days of growth, large root surface area was observed in P-replete than P-deficient large and medium patches when the patch strength was high (Fig. 2 top panel; T1 and T2). However, root surface area did not differ between P-replete and P- deficient patches when the patch strength was moderate, but it was considerably higher in large patches than small patches, particularly in P-replete patches (Fig. 2 top panel; T5–T8). Plants initially grown in P-enriched patch produced larger root surface area in P-replete large and medium size patches than small patches (Fig. 2 top panel; T9 and T10). After 60 days of growth, root surface area was still larger in P-replete than P-deficient large patch with high patch strength while the reverse was observed when the patch strength was moderate (Supplementary material S2). Larger root surface area was still observed after 90 days of growth in P-replete than P-deficient medium size patches with high and moderate patch strength (Fig. 2 bottom panel; T2 and T6).

**Fig. 3:**
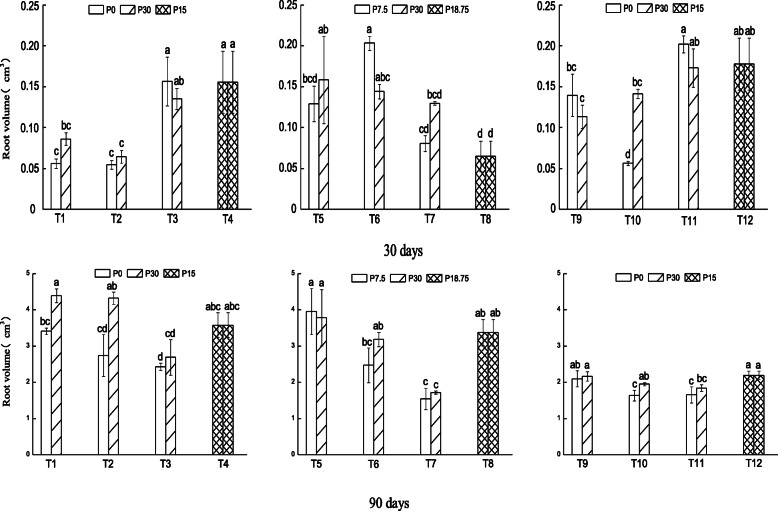
Root volume (cm^3^) of *N. reynaudiana* grown in high and low P patches under heterogeneous and homogenous P distribution for 30 (top panel) and 90 days (bottom panel). Treatments, T1 – T12, are as described in Fig. 1 and values are mean ± SE (*n* = 3), * indicates significant difference at *p* < 0.05 and lowercase letters indicate significant difference between treatments

Root volume of plants after 30 days of growth was larger in small patch than in large and medium patches when the patch strength was high, but no significant difference was observed between P-replete and P-deficient patches (Fig. 3 top panel; T1–T4). When the patch strength was moderate, root volume was larger in P-deficient than P-replete medium size patch, while it was larger in P-replete than P-deficient small size patch (Fig. 3 top panel; T6 and T7). Root volume was also larger in P-replete than P-deficient medium size patch in plants initially grown in P-enriched patch (Fig. 3 top panel; T10). After 60 days of growth, root volume was larger in P-replete than P-deficient large patch when the patch strength was high, while it was larger in P-deficient large patch when the patch strength was moderate (Supplementary material S3), but no significant difference was observed in plants initially grown in P-enriched patch. Root volume after 90 days of growth was larger in P-replete than P-deficient large and medium size patches when the patch strength was high (Fig. 3 bottom panel; T1 and T2). When the patch strength was moderate, root volume was generally larger in large than small patches (Fig. 3 bottom panel; T5–T8) while no significant difference was observed among levels of patch size in plants initially grown in P-enriched patches (Fig. 3 bottom panel; T9–T12).

**Fig. 4:**
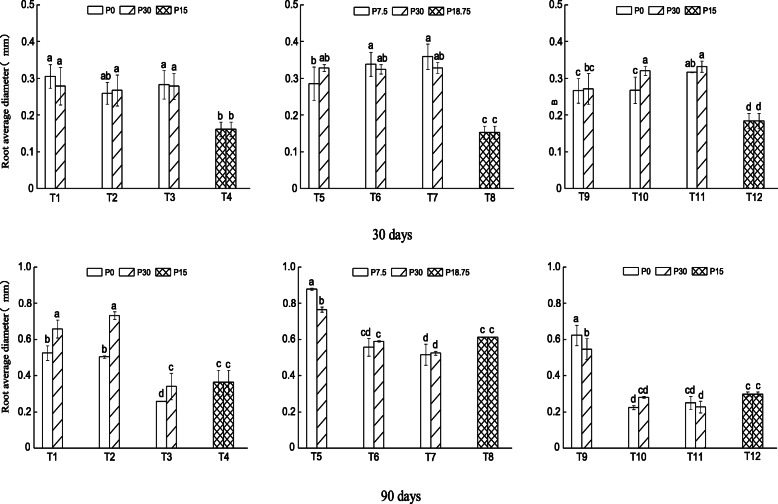
Average root diameter (mm) of *N. reynaudiana* grown in high and low P patches under heterogeneous and homogenous P distribution for 30 (top panel) and 90 days (bottom panel). Treatments, T1 – T12, are as described in Fig. 1 and values are mean ± SE (*n* = 3), * indicates significant difference at *p* < 0.05 and lowercase letters indicate significant difference between treatments

Average root diameter after 30 days of growth did not differ significantly between patches irrespective of the patch size and the patch strength, but it was higher for the heterogeneous than homogeneous P distribution (Fig. 4 top panel). Plants grown for 60 days had bigger average root diameter when grown in P-replete than P-deficient large patch with moderate patch strength (Supplementary material S4). Plants grown for 90 days had higher average root diameter in P-replete than P-deficient large and medium size patches with high patch strength (Fig. 4 bottom panel; T1 and T2), but it was lower in P-replete than P-deficient large patch with moderate patch strength (Fig. 4 bottom panel; T5). All root morphological traits were similar in all patches in the homogenous P supply treatments.

### Root dry mass

Root dry mass of plants grown in high patch strength (0 and 30 mg.kg^− 1^) was larger in large and medium size patches with high P than low P concentration whereas root dry mass did not differ between high P and low P patches when plants were grown under moderate patch strength (7.5 and 30 mg kg^− 1^) and in initially P-enriched patch as well as homogenous P supply treatments (Table 2). As a whole, the morphological plasticity in root dry mass was higher under heterogeneous than homogeneous P-distribution, particularly in large and medium patches when the patch strength was high; in medium patch when the patch strength was moderate; and in small patch when seedlings were grown in initially P-enriched patch (Table 2).

**Table 2:** Root dry mass and morphological plasticity (Mplast) after 90 days of growth in spatially heterogeneous and homogeneous P supply (mean ± SE). For each level of patch strength, means followed by different lower and upper case letter (s) across columns are statistically different

Patch strength	Patch size	Root Dry Mass (g)		
		Low P	High P	Mplast
0 + 0/30	Large	0.84 ± 0.29b	1.11 ± 0.18b	1.41 ± 0.5B
	Medium	0.71 ± 0.24ab	1.02 ± 0.01b	1.58 ± 0.6B
	Small	0.34 ± 0.07a	0.39 ± 0.04a	1.22 ± 0.3A
	Homogeneous	0.36 ± 0.07a	0.36 ± 0.07a	1.00 ± 0.0
0 + 7.5/30	Large	0.74 ± 0.11b	0.70 ± 0.19a	0.95 ± 0.3A
	Medium	0.59 ± 0.10ab	0.68 ± 0.01a	1.16 ± 0.2B
	Small	0.50 ± 0.09a	0.57 ± 0.15a	1.15 ± 0.2AB
	Homogeneous	0.53 ± 0.04ab	0,53 ± 0.04a	1.00 ± 0.0
7.5 + 0/30	Large	0.56 ± 0.07b	0.51 ± 0.16a	0.93 ± 0.4A
	Medium	0.37 ± 0.03a	0.41 ± 0.03a	1.13 ± 0.1A
	Small	0.25 ± 0.09a	0.32 ± 0.09a	1.60 ± 1.1B
	Homogeneous	0.40 ± 0.06a	0.40 ± 0.06a	1.00 ± 0.0

### P contents, translocation and P use efficiency

The root P content varied significantly (*P* < 0.01) between high P- and low P-patches irrespective of the patch strength and patch size, as well as for the first and second order interaction. In high patch strength, root P content was higher in large patches with high P concentration than in either medium or small patches (Table 3). Under moderate patch strength, the root P content did not differ among different patch sizes. When the plant was initially grown in P-enriched patch, the root P content was higher in medium size patches with low P concentration than large and small patches. As a whole, the physiological plasticity (Pplast) was higher than 1 although not significantly different in response to patch strength and patch size (Table 3).

**Table 3:** Root P content (mg/g dry mass) and physiological plasticity (Pplast) after 90 days of growth in spatially heterogeneous and homogeneous P supply (mean ± SE). For each level of patch strength, means followed by different lower and upper case letter (s) across columns are statistically different

Patch Strength	Patch Size	Root P Content (mg)		
		High P-Patch	Low P-Patch	Pplast
0 + 0/30	Large	0.79 ± 0.04b	0.67 ± 0.17a	1.26 ± 0.23A
	Medium	0.41 ± 0.01a	0.37 ± 0.08a	1.16 ± 0.18A
	Small	0.38 ± 0.03a	0.27 ± 0.03a	1.46 ± 0.22A
	Homogeneous	0.34 ± 0.04a	0.34 ± 0.05a	1.00 ± 0.00A
0 + 7.5/30	Large	0.47 ± 0.02a	0.47 ± 0.03a	0.98 ± 0.09A
	Medium	0.43 ± 0.08a	0.37 ± 0.03a	1.19 ± 0.17A
	Small	0.43 ± 0.07a	0.40 ± 0.03a	1.07 ± 0.11A
	Homogeneous	0.46 ± 0.02a	0.46 ± 0.02a	1.00 ± 0.00A
7.5 + 0/30	Large	0.87 ± 0.06a	0.84 ± 0.06ab	1.05 ± 0.14A
	Medium	0.94 ± 0.01a	1.07 ± 0.01c	0.87 ± 0.05A
	Small	1.02 ± 0.08a	0.98 ± 0.08bc	1.05 ± 0.13A
	Homogeneous	0.66 ± 0.01a	0.66 ± 0.01a	1.00 ± 0.00A

The leaf P content varied significantly with respect to patch strength (*p* < 0.01), patch size (*p* = 0.01) and their interaction (*p* = 0.004). The leaf P content was lower in plants grown in initially P-enriched patch than in patches with high and moderate patch strength (Table 4). The leaf P content was higher in large and medium patches when the concentration gradient between patches was high than small patches and under homogeneous P supply. Similarly, stem P content varied significantly with respect to patch strength (*p* < 0.001), patch size (*p* < 0.001) and their interaction (*p* < 0.001). Stem P content was lower in plants grown under initially P-enriched patch than in both high and moderate patch strengths (Table 4). When the concentration gradient between patches was high, stem P was high in large patch than medium and small patches while it was higher in medium patch than high and small patches when the concentration gradient was moderate.

**Table 4:** P content (mg), P translocation (%) and P use efficient of *Neyraudia reynaudiana* after 90 days of growth in spatially heterogeneous and homogeneous P supply (mean ± SE). For each level of patch strength, means followed by different letter across within the column are statistically different

Patch Strength	Patch Size	Leaf P (mg)	Stem P (mg)	P-Translocation (%)	P Use-Efficiency
0 + 0/30	Large	9.31 ± 0.66b	4.95 ± 0.03c	90.8 ± 1.05a	0.92 ± 0.04b
	Medium	8.69 ± 0.71b	2.99 ± 0.15b	93.6 ± 1.19a	1.00 ± 0.05b
	Small	6.03 ± 0.11a	1.41 ± 0.08a	91.9 ± 0.16a	0.76 ± 0.01a
	Homog.	6.23 ± 0.05a	2.79 ± 0.29b	93.1 ± 0.67a	0.87 ± 0.01ab
0 + 7.5/30	Large	11.09 ± 0.31a	3.26 ± 0.28a	93.8 ± 0.59ab	1.01 ± 0.07a
	Medium	10.58 ± 1.16a	7.28 ± 0.87b	95.7 ± 0.08b	0.76 ± 0.05a
	Small	11.52 ± 0.27a	3.74 ± 0.31a	94.8 ± 0.35ab	0.78 ± 0.04a
	Homog.	9.06 ± 0.68a	3.10 ± 0.71a	92.9 ± 0.61a	0.87 ± 0.09a
7.5 + 0/30	Large	0.48 ± 0.06b	0.56 ± 0.04b	10.6 ± 0.32b	0.82 ± 0.06a
	Medium	0.39 ± 0.02ab	0.37 ± 0.02a	9.6 ± 0.24ab	1.30 ± 0.10b
	Small	0.25 ± 0.07a	0.25 ± 0.05a	9.4 ± 0.41ab	1.76 ± 0.13c
	Homog.	0.26 ± 0.02a	0.40 ± 0.04a	8.8 ± 0.11a	0.84 ± 0.08a

The P translocation to the shoots and P use efficiency also varied significantly with respect to patch strength (*p* < 0.001), patch size (*p* < 0.05) and their interaction (*p* < 0.001). Translocation of P to the shoots was much lower in plants grown under initial P-enriched patch followed by high patch strength than those grown in moderate patch strength (Table 4). However, the P use efficiency was higher when the plant grew in initially P-enriched patch followed by high concentration gradient in small patches than large and medium patches (Table 4).

### Biomass production

Total root dry mass, shoot dry mass, total plant dry mass and root to shoot dry mass ratio varied significantly (*p* < 0.05) with respect to patch strength, patch size and interaction between patch strength and patch size. Total root dry mass after 90 days of growth was significantly higher in large patch than small patch and homogeneous P distribution across all concentration gradient between patches, except moderate patch strength (Table 5). Shoot dry mass was higher in large and medium patch than small patch and homogenous P supply for seedlings grown under conditions of high (0 and 30 mg.kg^− 1^) and moderate (7.5 and 30 mg.kg^− 1^) P concertation gradient between patches. For plants initially grown in P-enriched patch (7.5 + 0 and 30 mg.kg^− 1^), shoot dry mass was higher in large than medium and small patches, but statistically similar with those grown under homogenous P supply. The total plant dry mass after 90 days of growth was higher in large patches than small patches (Table 6). The root to shoot dry mass ratio was larger in large and medium patches than small patch and homogenous P distribution (Table 6) when the concentration gradient was high (0 and 30 mg.kg^− 1^). As a whole, whole-plant biomass production was responsive to the spatial distribution of P as compared to homogeneous P distribution, as evidenced from statistically significant difference (*p* < 0.05) in sensitivity index across different P concentration gradients and patch size (Table 6). Plants grown under high concentration gradient between patches produced more biomass than those grown under homogenous P supply, particularly in large and medium patches.

**Table 5:** Total root and shoot dry mass (g) after 30, 60 and 90 days of growth of *N. reynaudiana* seedlings in spatially heterogeneous and homogeneous P supply (mean ± SE). For each level of patch strength, means followed by different letter across columns are statistically different

Patch attributes		Dry mass, 30 days		Dry mass, 90 days	
Patch Strength	Patch Size	Root	Shoot	Root	Shoot
0 + 0/30	Large	0.02 ± 0.002a	0.36 ± 0.09b	2.0 ± 0.4b	13.0 ± 0.2d
	Medium	0.01 ± 0.003a	0.20 ± 0.02a	1.7 ± 0.2b	11.7 ± 0.2c
	Small	0.01 ± 0.006a	0.31 ± 0.02b	0.7 ± 0.03a	5.6 ± 0.1a
	Homog.	0.01 ± 0.002a	0.32 ± 0.03b	0.7 ± 0.1a	7.9 ± 0.5b
0 + 7.5/30	Large	0.03 ± 0.005b	0.39 ± 0.02c	1.4 ± 0.2a	14.5 ± 1.0c
	Medium	0.02 ± 0.001ab	0.28 ± 0.02b	1.3 ± 0.1a	13.4 ± 0.4c
	Small	0.02 ± 0.003ab	0.25 ± 0.03b	1.1 ± 0.2a	11.9 ± 0.2b
	Homog.	0.01 ± 0.001a	0.16 ± 0.03a	1.1 ± 0.1a	10.3 ± 0.4a
7.5 + 0/30	Large	0.03 ± 0.005b	0.25 ± 0.04a	1.1 ± 0.1c	10.1 ± 0.6b
	Medium	0.02 ± 0.007a	0.22 ± 0.05a	0.8 ± 0.1b	8.1 ± 0.3b
	Small	0.03 ± 0.006b	0.38 ± 0.01b	0.6 ± 0.01a	6.1 ± 0.5a
	Homog.	0.02 ± 0.001a	0.24 ± 0.23a	0.8 ± 0.1b	9.0 ± 1.5b

**Table 6:** Root dry mass to shoot dry mass ratio and total dry mass together with sensitivity index after 90 days of growth of *N. reynaudiana* in spatially heterogeneous and homogeneous P supply (mean ± SE). For each level of patch strength, means followed by different letter across columns are statistically different

Patch Strength	Patch Size	Root:Shoot Ratio	Total Dry Mass (g)	Sensitivity Index
0 + 0/30	Large	0.15 ± 0.03b	14.99 ± 0.45d	1.75 ± 0.08b
	Medium	0.15 ± 0.02b	13.45 ± 0.21c	1.57 ± 0.13b
	Small	0.13 ± 0.01ab	6.37 ± 0.11a	0.74 ± 0.04a
	Homog.	0.09 ± 0.01a	8.59 ± 0.66b	
0 + 7.5/30	Large	0.10 ± 0.01a	15.94 ± 1.19b	1.40 ± 0.15b
	Medium	0.09 ± 0.004a	14.72 ± 0.52b	1.30 ± 0.08ab
	Small	0.09 ± 0.02a	12.95 ± 0.46a	1.14 ± 0.06a
	Homog.	0.10 ± 0.01a	11.37 ± 0.47a	
7.5 + 0/30	Large	0.11 ± 0.01b	11.18 ± 0.63c	1.16 ± 0.22b
	Medium	0.10 ± 0.004ab	8.87 ± 0.36b	0.92 ± 0.18ab
	Small	0.09 ± 0.01ab	6.64 ± 0.49a	0.68 ± 0.10a
	Homog.	0.09 ± 0.001a	9.84 ± 1.61bc	

## Discussion

The results confirm our first hypothesis that selective deployment of roots occurs in high P patches than low P patches; thus root morphological plasticity plays an adaptive role in foraging heterogeneous P distribution by this species. The observed root morphological response is significantly modulated by patch strength and size; i.e. the greater the patch strength and the larger the patch size, the greater the deployment of root in high P than low P patches would be. The significantly higher root morphological traits on the high P side in the split-P treatment imply that P-deficiency signal from the low P side might stimulate the growth of the roots located in the high-P zone. This rooting characteristics increase the chance of encountering nutrient-rich patches, thereby enabling plants to efficiently forage in a heterogeneous soil profile [25]. The increase in root morphological traits over time with slight differences among levels of patch strength and patch size is mainly ontogenic difference but suggests that the plant adjusts its root system to meet the P demand as growth advances. It could also be that the smaller size of the patch restricts growth of the roots mainly due to growing space limitation.

Spatial heterogeneity of nutrient in soils is reflected in the scale of the distribution of nutreints. During the growth and development process, plant roots experience nutrient patches of different scales, and plant roots make corresponding morphological response [26]. Studies have shown that plant roots have a threshold for the “perception” of nutrient heterogeneity scale [27]. Under the condition of small heterogeneity scale, plants ignore the heterogeneity and regard it as homogeneous, thus roots do not make plastic changes. However, when the heterogeneity scale is high enough, the root system cannot ignore the differences between nutrient patches in heterogeneous environments; thus a series of responses is triggered [27]. Studies have shown that the total root length in large patches can be three times higher than that in medium and small patches, and local root proliferation disappeared in small and medium patches under low P availability [1, 28–30]. Rhizomorphous clonal plants, like *N. reynaudiana*, obtain nutrient resources by branching outwards in large patches with high nutrient content, while those in small patches dispersed [31, 32]. This is in line with our findings that the root length, root surface area, root volume, and average root diameter in large patches were significantly higher than those in small patches.

In addition, the increased placement of roots in large patches with higher P concentration gradient could be related to the carbon cost for increased production of roots in smaller patches with low concentration gradient. Consequently, the plant allocates more resource to produce more roots in large patches to optimize nutrient capture. Interestingly, all root morphological traits were low when the plant was initially grown in P-enriched patches than without initially encountering P (T9–T11 in Figs. 1, 2, 3 and 4). This indicates that *N. reynaudiana* is content with small amount of initial P availability, but slowly proliferated its root to forage patchily distributed P. As a whole, our results are consistent with previous studies that had demonstrated increased root deployment in localized nutrient-rich patches i.e., root morphological plasticity, for a range of other species [5, 7, 8, 11, 17, 33–35].

The results also confirmed our second hypothesis that increased root P contents in high P- than low P- patch; suggesting that physiological plasticity plays an adaptive role in foraging patchily distributed P in the soil by this species. Furthermore, the increased uptake of P in localized P-rich patches indicates that the species is highly efficient in resource acquisition. The fact that roots in patches with no P availability (0/30) had a certain amount of root P suggests internal redistribution of P to maintain P homeostasis. Similar results have been observed in low P tolerant Chinese fir genotypes [36]. The translocation of P to the shoots and P content of leaf and stem were lower in plants initially grown in P-enriched patch followed by high P concentration gradient between patches. However the P use efficiency was higher, especially in small patches. This indicates that this species has high P utilization efficiency when the availability of P is low. Utilization efficiency, defined as the amount of biomass per unit of nutrient present in the biomass, can be achieved through remobilization of internal P, increased activity of enzymes that replace P in structural compounds or during metabolism [37, 38], or reduced consumption of P [39].

Our result shows that total plant biomass is higher in heterogeneous than in homogeneous nutrient environment, but vary with patch strength and patch size, which confirms our third hypothesis. The total plant dry mass was significantly higher in large and medium patches in both high and low P concentration gradient between patches. However plants initially grown in P-enriched patches produced more dry mass in large than small patch and homogeneous P distribution. This is consistent with previous studies that demonstrated increased whole-plant productivity and growth rates in patchily nutrient distribution environment compared with homogeneous environment [10, 11, 33]. Generally, *N. reynaudiana* produced less root than shoots (i.e. low root to shoot ratio) across all treatments; suggesting that the species has high P utilization efficiency, as also observed for other species [40]. Shoot biomass production was favored by availability of high concentration of P in the growing media. This is not surprising as P is the main growth-limiting nutrient in the study area while it is essential for various plant metabolic processes. As a whole, biomass production is more responsive to heterogeneous than homogeneous P supply.

## Conclusions

The findings demonstrate that foraging strategy for patchily distributed nutrients in soils is highly dependent on both patch strength and patch size. *N. reynaudiana* efficiently forages patchily distributed P through deployment of more roots in P-replete patches than P-deficient patches, and larger difference in patch strength and patch size induces deployment of more root in localized P-replete patches. The increased deployment of root in P-replete patches resulted in increased P content in roots, leaves and stem, suggesting that physiological plasticity plays an adaptive role in foraging patchily distributed P, depending on patch size and strength. Thus, both root morphological plasticity and physiological plasticity are the main adaptation mechanisms for foraging patchily distributed P by this species. Efficient foraging of P in spatially heterogeneous environment resulted in increased total plant biomass production; thus biomass production is more responsive to heterogeneous than homogeneous P distribution.

## Methods

### Experimental material

*N. reynaudiana* seedlings were used as experimental material to investigate their foraging strategy to spatially heterogeneous P distribution. Seeds of *N. reynaudiana* were purchased from a company in Kunming, Yunnan Province (Yunnan Jinye Eco-construction Group Co., Ltd) and sown in climate chamber set at 25 °C, 75% relative humidity, and a photoperiod of 12 h light (photon flux density 4000 lx) and 12 h dark. After 2 weeks, the germinants were transferred to nutrient-rich humus substrate and left to grow in a greenhouse set at 29.3 °C/23 °C (day/night); photon flux density of 21 mol quanta m^− 2^ d^− 1^, and ca. 42.7 and 67.7% relative humidity during the light and dark periods of the experiment, for 4 weeks. Seedlings with relatively uniform size (12 cm ± 1 cm in height, 4 cm ± 1 cm in root length, and 10 mg ± 1.5 mg in fresh weight) were selected for this experiment.

### Experimental design and growth conditions

**Fig. 5:**
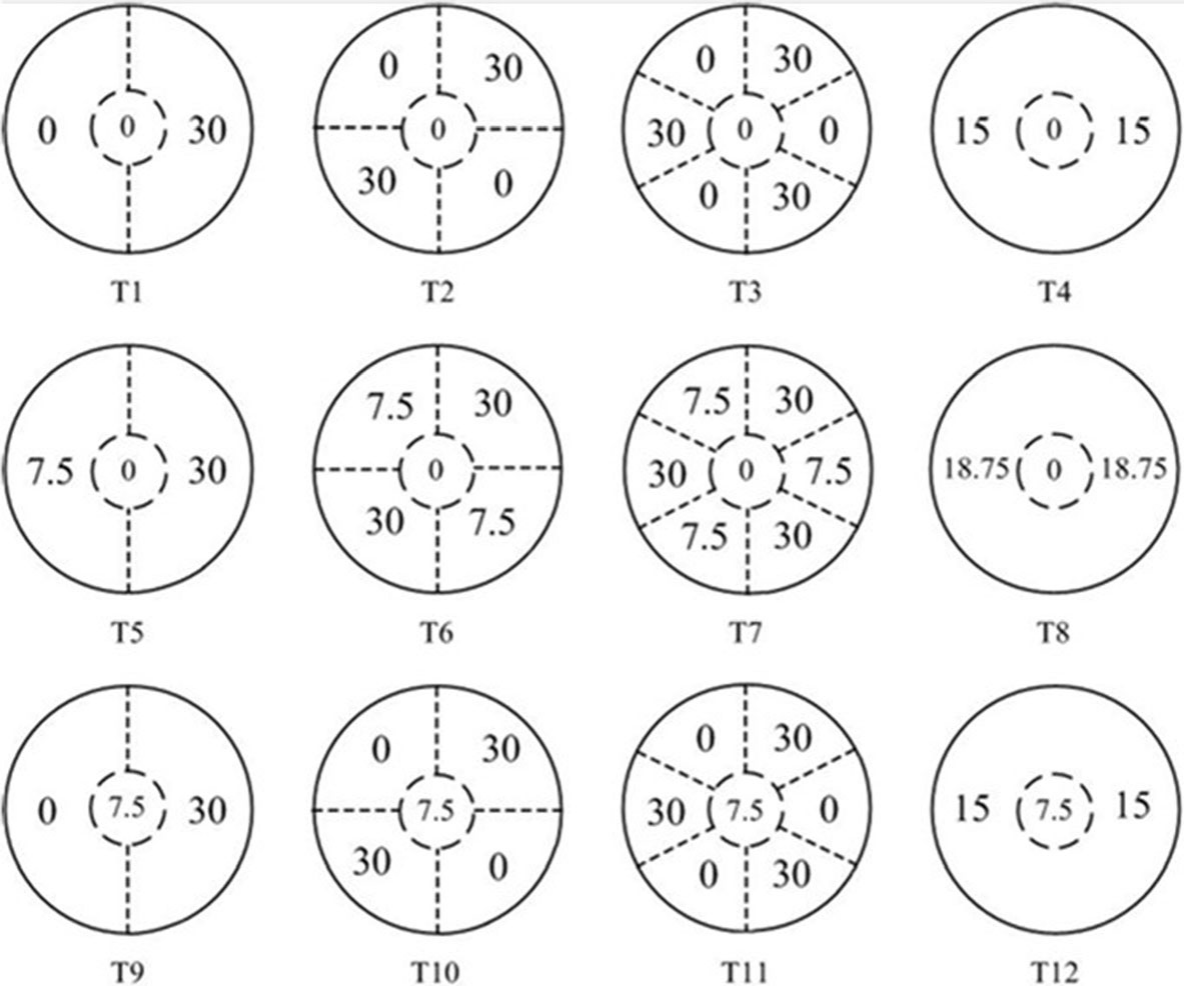
Sketch of the experimental pot used to simulate patchily P distribution, and experimental design showed the size and the amount of P nutrient (mg· P kg^− 1^). The values 0, 7.5, 15, 18.75, 30 showed the amount of P added in each patch. T1, T5, T9 indicated large patch, T2, T6, T10 indicated medium patch, T3, T7, T11 indicated small patch with different P concentrations, and T4, T8、T12 indicated homogeneous P distribution with different P concentrations

To investigate the foraging strategy of *N. reynaudiana* seedlings to spatially heterogeneous P supply, a factorial experiment, which involved three levels of patch strength (0 + 0/30, 0 + 7.5/30 and 7.5 + 0/30 mg P. kg^− 1^) and three levels of patch size (small, medium and large) were established (Fig. 5). The treatment 0 + 0/30 involved no P addition in the central interior patch where the seedlings were initially planted and intended to simulate the effect of high patch strength on root proliferation in the exterior P-deficient and P-replete patches. Similarly, the treatment 0 + 7.5/30 were intended to simulate the effects of moderate patch strength on root proliferation in the exterior P-deficient and P-replete patches. The treatment 7.5 + 0/30 mg P. kg^− 1^ involved addition of small amount of P in the central interior patch and intended to see if root proliferation between P-deficient and P-replete patches was influenced by initial P encounter. In addition, a homogeneous P supply with three levels (15/15, 18.75/18.75 and 15/15 + 7.5 mg P. kg^− 1^ per patch), corresponding to the total P concentration in heterogeneous P supply, was included to compare total biomass production between heterogeneous and homogeneous P supply. The P concentrations used in this study were determined based on P availability in the soil in southern China. A total of 12 treatments were applied in the experiment: 9 heterogeneous and 3 homogenous P treatments.

The experiment was conducted using pots (diameter: 40 cm, height: 32 cm) that were divided into ½, ¼ and 1/6 baffle less compartments using plastic separator to simulate large, medium and small patches, respectively. Each pot compartment was filled with a mixture of sand and sodium polyacrylate prior to application of different P concentrations as the sodium polyacrylate (0.5–1 mm in diameter) strongly adsorbed the applied P and hence prevented its movement between compartments but capable of releasing nutrients slowly and evenly and could be freely penetrated by roots. According to the weight of each pot, the sodium polyacrylate and washed river sand, the different concentrations of KH_2_PO_4_ solution (as P source) were mixed and packed in a volume ratio of 1:3 in each pot. In each treatment pot, one *N. reynaudiana* seedling was planted in a cylindrical tube (30 cm in length and 8 cm in diameter) filled with sand or sand mixed with 7.5 mg KH_2_PO_4_·kg^− 1^ at the center of the pot. Once the plants were placed correctly at the center of the treatment pots, the cylindrical tube and patch separators were removed carefully (Fig. 5). Each treatment had three replicates of independent plants.

The experiment was carried out in the greenhouse of the Forestry College, Fujian Agriculture and Forestry University under the following environmental conditions: 29.3 °C/23 °C (day/night); photon flux density of 21 mol quanta m^− 2^ d^− 1^, and ca. 42.7 and 67.7% relative humidity during the light and dark periods of the experiment, respectively. Seedlings were left to grow under this condition for 3 months. To meet the growth requirements for other nutrients, 50 mL of nutrient solution adjusted to a pH of 5.5 was supplied to each pot compartment every 5 days. Macro-nutrients were supplied according to a modified Hoagland solution as 0.51 gL^− 1^ KNO_3_, 0.82 gL^− 1^ Ca (NO_3_)_2_, 0.49 gL^− 1^ MgSO_4_•7H_2_O and 0.136 gL^− 1^ KCl (Chen et al. 1992). Micro-nutrients were also supplied according to Amon formula as 2.86 gL^− 1^ H_3_BO_3_, 0.08 gL^− 1^ CuSO_4_•5H_2_O, 0.22 gL^− 1^ ZnSO_4_•7H_2_O, 1.81 gL^− 1^ MnCl_2_•4H_2_O, 0.09 gL^− 1^ H_2_MoO_4_•H_2_O and 20 gL^− 1^ Fe^2+^EDTA. The seedlings were watered every two to 3 days depending on the moisture content of the substrate.

### Measurement of root morphological traits

To examine temporal variation in root morphological traits, plants were harvested after 30, 60 and 90 days of treatment application by first draining down the sand from each pot with water, and then the roots were tied up in bundle from each compartment in both homogeneous and heterogeneous P treatments and thereafter carefully pulled out each seedling. All fine roots were collected from the growing media in each compartment. The roots from the different compartments were cleaned with distilled water separately, quickly dried with paper towels, and scanned with digital scanner (STD1600 Epson USA) with non-overlapping tiles. The root morphological traits (total root length, total root surface area, total root volume and average root diameter) from each compartment were determined by WinRhizo root analysis system (Version 4.0 B; Regent Instruments Inc., Canada).

### Analysis of P contents

After 90 days of growth, plants were harvested and the P concentration in leaves, stems and roots were determined by digesting 1.0 g of dry plant material in 10 mL of acid mixture (H_2_SO_4_:HClO_4_ 10:1). Phosphorus was determined by the molybdenum-blue colorimetric method. For each replicate, three independent samples were analyzed and the average value recorded. P content was computed by multiplying P concentration of the sample by dry mass of the respective organ; P translocation was computed as a ratio of shoot P to total P; and P use efficiency was calculated as the ratio of shoot dry mass to shoot P.

### Analysis of biomass production

To determine dry matter of roots, shoots (stems and leaves) and whole-plant biomass, the shoots and roots of the harvested plants were oven-dried first at 105 °C for 30 min and then at 79 °C until constant mass. Then the dry mass of roots and shoots were determined using sensitive balance (with precision of 0.0001 g). Whole-plant biomass was calculated as sum of root and shoot dry masses.

### Statistical analysis

To examine the foraging strategy of *N. reynaudiana* seedlings to spatially heterogeneous P supply, four-way between-groups multivariate analysis of variance (MANOVA) was performed using time, patch strength, patch size and high P versus low P compartments as fixed independent variables while taking root morphological traits as dependent variables. The inflation of Type 1 error was controlled by Bonferonni adjustment for multiple comparisons (Quinn and Keough, 2002). With four treatments in our study, time, patch strength, patch size and high versus low P compartments, six possible pair-wise comparisons could be made, thus the Bonferonni adjusted *p* value was 0.0083 (0.05/6). Results of the statistical analyses were considered significant if *p* < 0.0083 and to show tendencies if 0.0083 < *p* < 0.05. Three-Way ANOVA was performed to examine differences in root dry mass and root P contents with respect to patch strength, patch size and between P-replete and P-deficient patches while Two-ANOVA was performed to determine the effects of patch strength and patch size on leaf and stem P contents, P translocation and P use efficiency.

To examine the foraging behavior and its benefit in whole-plant biomass production, root morphological plasticity (Mplast), root physiological plasticity (Pplast) and sensitivity index (SI) were calculated for each level of patch strength and patch size. Mplast is increased root proliferation in nutrient-rich patch than in nutrient-poor patch while Pplast is increased rate of nutrient uptake in the nutrient-rich patch than nutrient-poor patch [11, 18, 41]. Thus, Mplast and Pplast were calculated following Mou et al. [42] and Zhang et al. [11] as:

$$ Mplast=\frac{\left( Rdm\  in\ high\ P\  patch\right)-\left( Rdm\  in\  low\ P\  patch\right)}{Total\  Rdm} $$

$$ Pplast=\frac{RP\  in\ high\ P\  patch}{RP\  in\  low\ P\  patch} $$

Where Rdm and RP were root dry mass and root P contents, respectively. For the homogeneous treatment, Mplast and Pplast were computed as the ratio of root dry mass/root P content in two opposite patches. Mplast and Pplast are expected to be one in the homogenous treatment, but greater than one in the heterogeneous treatment.

SI, as an index of how the total plant biomass responded to heterogeneous P supply compared to homogenous P supply [11], was computed as the ratio of plant total biomass (total root dry mass and shoot dry mass) in the heterogeneous treatment to that in the homogenous treatment for each level of patch strength and patch size. The following formula was used to calculate SI:

$$ SI=\frac{TotBm\ in\ hetero}{TotBm\ in\ homo} $$

Where TotBm in hetero and homo stands for total biomass in heterogeneous and in homogenous P distribution environment, respectively.

Two-way ANOVA was performed to examine the effects of patch strength and patch size on shoot (leaf + stem) dry mass and total biomass (shoot + root), Mplast, Pplast and SI. Means that showed significant differences were compared by Tukey’s Post hoc test (*p* < 0.05). All statistical analyses were computed using SPSS Statistical Package (SPSS 20.0, SPSS Ins., Chicago, IL, U.S.A.).

## Supplementary Information

**Additional file 1.** Supplementary material.

## Data Availability

The datasets used and/or analyzed during the current study are available from the corresponding author on reasonable request.

## Abbreviations

P: Phosphorus
N: Nitrogen
Mplast: Root morphological plasticity
Pplast: Root physiological plasticity
SI: Sensitivity index

## References

[1] Liu YJ, Bortier MF, De Boeck HJ, Nijs I (2017). Root distribution responses to three-dimensional soil heterogeneity in experimental mesocosms. Plant Soil.

[2] McDowell RW, Hill SJ (2015). Speciation and distribution of organic phosphorus in river sediments: a national survey. J Soils Sediments.

[3] Jackson RB, Caldwell MM (1993). The scale of nutrient heterogeneity around individual plants and its quantification with geostatistics. Ecology.

[4] Farley RA, Fitter AH (1999). Temporal and spatial variation in soil resources in deciduous woodland. J Ecol.

[5] Wang L, Mou P, Jones RH (2006). Nutrient foraging via physiological and morphological plasticity in three plant species. Can J For Res.

[6] Nie YP, Chen HS, Wang KL, Ding YL (2014). Rooting characteristics of two widely distributed woody plant species growing in different karst habitats of Southwest China. Plant Ecol.

[7] He Y, Liao H, Yan X (2003). Localized supply of phosphorus induces root morphological and architectural changes of rice in split and stratified soil cultures. Plant Soil.

[8] Hou X, Tigabu M, Zhang Y, Ma X, Cai L, Wu P, Liu AQ, Wang C, Qiu H (2017). Root plasticity, whole plant biomass, and nutrient accumulation of Neyraudia reynaudiana in response to heterogeneous phosphorus supply. J Soils Sediments.

[9] Karthikeyan AS, Jain A, Nagarajan VK, Sinilal B, Sahi SV, Raghothama KG (2014). Arabidopsis thaliana mutant lpsi reveals impairment in the root responses to local phosphate availability. Plant Physiol Biochem.

[10] Hutchings MJ, Wijesinghe DK (2008). Performance of a clonal species in patchy environments: effects of environmental contention yield at local and whole-plant scales. Evol Ecol.

[11] Zhang Y, Zhou Z, Ma X, Jin G (2010). Foraging ability and growth performance of four subtropical tree species in response to heterogeneous nutrient environments. J For Res.

[12] Dunbabin V, Rengel Z, Diggle A (2001). Lupinus angustifolius has a plastic uptake response to heterogeneously supplied nitrate while Lupinus pilosus does not. Aust J Agric Res.

[13] Drew MC (1975). Comparison of the effect of a localized supply of phosphate, nitrate, ammonium and potassium on the growth of the seminal root system, and the shoot, in barley. New Phytol.

[14] Jackson RB, Pockman WT, Hoffmann WA, Pugnaire FI, Valladares F (1999). The structure and function of root systems. Hand book of functional plant ecology.

[15] Proe MF, Millard P (1995). Effect of P supply upon seasonal growth and internal cycling of P in Sitka spruce Picea sitchensis (bong.) Carr. Seedlings. Plant Soil.

[16] Hodge A (2004). The plastic plant: root responses to heterogeneous supplies of nutrients. New Phytol.

[17] Kume T, Sekiya N, Yano K (2006). Heterogeneity in spatial P-distribution and foraging capability by Zea mays: effects of patch size and barriers to restrict root proliferation within a patch. Ann Bot.

[18] Mou P, Jones RH, Tan Z, Bao Z, Chen H (2013). Morphological and physiological plasticity of plant roots when nutrients are both spatially and temporally heterogeneous. Plant Soil.

[19] Wang YZ, Cai LP, Zhou CF, Hou XL, Zou XH (2017). Review of stress resistance and application of the pioneer plant Neyraudia reynaudiana. Pratac Sci.

[20] Cai LP, Wu PF, Hou XL, Ma XQ, Jiang S, Ren JJ (2012). Effects of phosphorus stress on photosynthetic characteristics of pioneer plant Neyraudia reynaudiana on soil and water conservation. J Soil Water Conserv.

[21] Feng H, Guo YB, Lin RQ, Peng GX, Tan ZY (2009). Preliminary studies on endophytic diazotrophs from Neynaudia reynaudiana and Cynodon dactyion. J Trop Subtrop Botany.

[22] Yang ST (2008). Analysis on the relationship between the growth of the Neyraudia reynaudiana and the VA mycorrhiza. Subtrop Soil Water Conserv.

[23] Feng H, Li YT, Zhang ZH, Wei XH, Guo YB (2010). Screening, identification, and capability assessment of a phosphorus solubilizing fungus in rhizosphere of Burma reed. Microbiol China.

[24] Cai LP (2012). The mechanism of eco-physiological response to environmental stress for pioneer plant *Neyraudia reynaudiana* in collapsing hill area.

[25] Wijesing DK, John EA, Beurskens S, Hutchings MJ (2001). Root system size and precision in nutrient foraging: responses to spatial pattern of nutrient supply in six herbaceous species. J Ecol.

[26] Wei HX, Guo P, Zheng HF, He XY, Wang PJ, Ren ZB, Zhai C (2017). Micro-scale heterogeneity in urban forest soils afects? Ne root foraging by ornamental seedlings of Buddhist pine and Northeast yew. Urban Forestry Urban Greening.

[27] Henke M, Sarlikioti V, Kurth W, Buck-Sorlin GH, Pages L (2014). Exploring root developmental plasticity to nitrogen with a three-dimensional architectural model. Plant Soil.

[28] Yano K, Kume T (2006). Root morphological plasticity for heterogeneous phosphorus supply in Zea mays L. Plant Prod Sci.

[29] Gao Y, Xing F, Jin YJ, Nie DD, Wang Y (2012). Foraging responses of clonal plants to multi-patch environmental heterogeneity: spatial preference and temporal reversibility. Plant Soil.

[30] McNickle GG, Brown JS. When Michaelis and Menten met Holling: towards a mechanistic theory of plant nutrient foraging behavior. AOB Plants. 2014. 10.1093/aobpla/plu066. 10.1093/aobpla/plu066 PMC4271705 25341427

[31] Peng YK, Luo FL, Li HL, Yu FH (2013). Growth responses of a rhizomatous herb Bolboschoenus planiculmis to scale and contrast of soil nutrient heterogeneity. Chinese J Plant Ecol.

[32] Qian YQ, Luo D, Gong G, Han L, Ju GS, Sun ZY (2014). Effects of spatial scale of soil heterogeneity on the growth of a clonal plant producing both spreading and clumping ramets. J Plant Growth Regul.

[33] Nakamura R, Kachi N, Suzuki JI (2008). Root growth and plant biomass in Lolium perenne exploring a nutrient-rich patch in soil. J Plant Res.

[34] Mommer L, Visser EJW, Ruijven J, Caluwe H, Pierik R, Kroon H (2011). Contrasting root behavior in two grass species: a test of functionality in dynamic heterogeneous conditions. Plant Soil.

[35] Zhou C, Jiang W, Li Y, Hou X, Liu AQ, Cai L (2017). Morphological plasticity and phosphorus uptake mechanisms of hybrid Eucalyptus roots under spatially heterogeneous phosphorus stress. J For Res.

[36] Farooq TH, Tigabu M, Ma XQ, Zou XH, Liu AQ, Odén PC, Wu P (2018). Nutrient uptake, allocation and biochemical changes in two Chinese fir cuttings under heterogeneous phosphorus supply. iForest Biogeosci Forestry.

[37] Li M, Welti R, Wang X (2006). Quantitative profiling of Arabidopsis polar glycerolipids in response to phosphorus starvation. Roles of phospholipases Dz1 and Dz2 in phosphatidylcholine hydrolysis and digalactosyldiacylglycerol accumulation in phosphorus-starved plants. Plant Physiol.

[38] Hammond JP, White PJ (2008). Sucrose transport in the phloem: integrating root responses to phosphorus starvation. J Exp Bot.

[39] Shenoy VV, Kalagudi GM (2005). Enhancing plant phosphorus use efficiency for sustainable cropping. Biotechnol Adv.

[40] Wu P, Tigabu M, Ma XQ, Odén PC, He Y, Yu X, He Z (2011). Variations in biomass, nutrient contents and nutrient use efficiency among Chinese fir provenances. Silvae Genetica.

[41] Johnson HA, Biondini ME (2001). Root morphological plasticity and nitrogen uptake of 59 plant species from the Great Plains grasslands, U.S.A. Basic Appl Ecol.

[42] Mou P, Mitchell RJ, Jones RH (1997). Root distribution of two tree species under a heterogeneous nutrient environment. J Appl Ecol.

