# Supplementary material for: Strength and size of phosphorus-rich patches determine the foraging strategy of Neyraudia reynaudiana
Source: BMC Plant Biol. 2020 Dec 7;20:545. doi: 10.1186/s12870-020-02738-0 (PMC7720531; doi:10.1186/s12870-020-02738-0)
Supplement: Supplementary file 1 — Additional file 1. Supplementary material. [file 12870_2020_2738_MOESM1_ESM.docx]

Supplementary material

S1. Total root length (m) of *N. reynaudiana* grown in high and low P patches under heterogeneous and homogenous P distribution for 60 days where T1, T2 and T3 had high P concentration gradient between patches (0 and 30 mg.kg^−1^), T5, T6 and T7 had moderate P concentration gradient (7.5 and 30 mg kg^−1^), T9, T10 and T11 had initial P concentration of 7.5 mg kg^−1^ in the central interior patch, and subsequently high P concentration gradient between patches (0 and 30 mg kg^−1^); and T4, T8 and T12 had homogenous P distribution of 15, 18.5 and 7.5 + 15 mg kg^−1^, respectively. Values are mean ± SE (n = 3), * indicates significant difference at *p* ＜ 0.05 and lowercase letters indicate significant difference between treatments.

S2. Root surface area (cm^2^) of *N. reynaudiana* grown in high and low P patches under heterogeneous and homogenous P distribution for 60 days where T1, T2 and T3 had high P concentration gradient between patches (0 and 30 mg.kg^−1^), T5, T6 and T7 had moderate P concentration gradient (7.5 and 30 mg kg^−1^), T9, T10 and T11 had initial P concentration of 7.5 mg kg^−1^ in the central interior patch, and subsequently high P concentration gradient between patches (0 and 30 mg kg^−1^); and T4, T8 and T12 had homogenous P distribution of 15, 18.5 and 7.5 + 15 mg kg^−1^, respectively. Values are mean ± SE (n = 3), * indicates significant difference at *p* ＜ 0.05 and lowercase letters indicate significant difference between treatments.

S3. Root volume (cm^3^) and average root diameter (mm) of *N. reynaudiana* grown in high and low P patches under heterogeneous and homogenous P distribution for 60 days where T1, T2 and T3 had high P concentration gradient between patches (0 and 30 mg.kg^−1^), T5, T6 and T7 had moderate P concentration gradient (7.5 and 30 mg kg^−1^), T9, T10 and T11 had initial P concentration of 7.5 mg kg^−1^ in the central interior patch, and subsequently high P concentration gradient between patches (0 and 30 mg kg^−1^); and T4, T8 and T12 had homogenous P distribution of 15, 18.5 and 7.5 + 15 mg kg^−1^, respectively. Values are mean ± SE (n = 3), * indicates significant difference at *p* ＜ 0.05 and lowercase letters indicate significant difference between treatments.

S4. Average root diameter (mm) of *N. reynaudiana* grown in high and low P patches under heterogeneous and homogenous P distribution for 60 days where T1, T2 and T3 had high P concentration gradient between patches (0 and 30 mg.kg^−1^), T5, T6 and T7 had moderate P concentration gradient (7.5 and 30 mg kg^−1^), T9, T10 and T11 had initial P concentration of 7.5 mg kg^−1^ in the central interior patch, and subsequently high P concentration gradient between patches (0 and 30 mg kg^−1^); and T4, T8 and T12 had homogenous P distribution of 15, 18.5 and 7.5 + 15 mg kg^−1^, respectively. Values are mean ± SE (n = 3), * indicates significant difference at *p* ＜ 0.05 and lowercase letters indicate significant difference between treatments.

S1.

S2

S3

S4
